# A CASE REPORT OF DAPAGLIFLOZIN ASSOCIATED PANCREATITIS

**DOI:** 10.51894/001c.123051

**Published:** 2024-08-30

**Authors:** Chaoneng Wu

**Affiliations:** 1 Internal Medicine Garden City Hospital

## 67

### BACKGROUND

Diabetes mellitus (DM) poses a global health threat and is a leading cause of death with substantial health costs. Sodium-glucose cotransporter-2 inhibitors (SGLT2i) are a newer class of antidiabetic drugs, including Dapagliflozin, Canagliflozin, Empagliflozin, and others. This case highlights Dapagliflozin’s association with pancreatitis.

### CLINICAL SIGNIFICANCE

SGLT2i, with high selectivity for SGLT2 in renal proximal tubules, exhibit significant effects on glycated hemoglobin (HbA1c) reduction, blood pressure, and weight loss. Glucose lowering effect is diminished if the eGFR is <45 mL per min per 1·73 m². Canagliflozin has been approved for patients with eGFR <30 mL per min per 1·73 m² because of renal protective benefits. However, adverse effects, including rare events like Fournier’s gangrene, diabetic ketoacidosis, and leg amputation, should be considered (Tang et al., 2020).

### REPUTED RANDOMIZED CONTROLLED TRIALS (RCTS)

Existing RCTs report no pancreatitis risk associated with SGLT2i. However, post-marketing surveillance remains crucial for real-time assessment.

### CASE DESCRIPTION

A 76-year-old female with Type 2 DM, obesity, and hypertension presented with one-week epigastric pain. Past history included a lipase elevation without evident cause one year ago. Current lipase was persistently elevated (178 U/L) despite improved HbA1c (8.1% to >7.0%). CT scan revealed acute pancreatitis without necrosis, and careful medication review identified that she was started on Dapagliflozin and Glimepiride 10 weeks ago in addition to her longstanding regimen of Metformin, Aspirin, Lovastatin, Amlodipine, and Lisinopril. Symptoms resolved upon discontinuation of Dapagliflozin.

### DISCUSSION

Drug-induced pancreatitis is rare (<5%), and SGLT2i association is documented in limited cases. This patient’s presentation aligns with acute pancreatitis after Dapagliflozin introduction, with no evidence of other causes. The mechanism behind Dapagliflozin-induced pancreatitis remains unclear but may involve idiosyncratic reactions or immunological effects.

### CONCLUSION

Patients on SGLT2i should be informed about potential pancreatitis risk, reporting symptoms promptly. Healthcare providers must be vigilant for pancreatitis signs among SGLT2i users, promptly discontinuing the suspected medication to minimize risks.

